# Coordination Driven Capture of Nicotine Inside a Mesoporous MOF

**DOI:** 10.3390/ma10070727

**Published:** 2017-06-30

**Authors:** Davide Balestri, Davide Capucci, Nicola Demitri, Alessia Bacchi, Paolo Pelagatti

**Affiliations:** 1 Dipartimento di Scienze Chimiche, della Vita e della Sostenibilità Ambientale, Università degli Studi di Parma, Parco Area delle Scienze 17/A, 43124 Parma, Italy; davide.balestri@studenti.unipr.it (D.B.); davide.capucci@libero.it (D.C.); alessia.bacchi@unipr.it (A.B.); 2 Elettra-Sincrotrone Trieste, S.S. 14 Km 163.5 in Area Science Park, 31149 Basovizza, Trieste, Italy; nicola.demitri@elettra.eu

**Keywords:** MOF, nicotine, crystalline sponge, inclusion, host-guest

## Abstract

Metal organic frameworks (MOFs) are a wide class of crystalline porous polymers studied in many fields, ranging from catalysis to gas storage. In the past few years, MOFs have been studied for the encapsulation of organic or organometallic molecules and for the development of potential drug carriers. Here, we report on the study of two structurally-related mesoporous Cu-MOFs, namely PCN-6 and PCN-6′ (PCN stands for Porous Coordination Network), for nicotine trapping. Nicotine is a well-known alkaloid liquid molecule at room temperature, whose crystalline structure is still unknown. In this work, the loading process was monitored by electron ionization mass spectrometry by using a direct insertion probe (DIP-EI/MS), infrared (IR), and ultraviolet/visible (UV/VIS) analysis. Both nuclear magnetic resonance (NMR) spectroscopy and thermogravimetric (TGA) analysis showed evidence that nicotine trapping reaches remarkable uptakes up to 40 wt %. In the case of PCN-6@nicotine, X-ray structural resolution revealed that the guest uptake is triggered by coordination of the pyridine ring of nicotine to the copper nuclei of the paddle-wheel units composing the framework of PCN-6.

## 1. Introduction

Nicotine is a natural chiral alkaloid which is contained in tobacco leaves, approximately 0.6–3.0% of the dry plant weight. It functions as a natural anti-herbivore agents in some Solanaceae, such as aubergine and tomato [1]. For this reason, until the middle of 20th century, nicotine found a broad use as an insecticide in different formulations [2,3]. However, the relative elevated cost compared to other synthetic pesticides and the high human toxicity associated with the absorption of high amounts of nicotine [4], an issue to which the operators assigned to its use were potentially exposed, strongly limited its application. As all smokers know, nicotine is highly addictive. As such, one of its main therapeutic uses is in the treatment of nicotine dependence [5], although it has been investigated for its stimulant properties, as well [6]. Since nicotine can be considered as a liquid active pharmaceutical ingredient (API), it is highly desirable to find some method to stabilize it in crystalline form. Liquids, in fact, are intrinsically less stable than solids and, indeed, most APIs are manufactured and distributed as crystalline forms. In order to reach this goal, we directed our attention on the use of metal organic frameworks, being inspired by the “crystallization sponge method” recently developed by Fujita [7]. Metal organic frameworks (MOFs) are a relatively new class of porous crystalline materials, deriving from the self-assembly of metal ions or metal aggregates (SBU = secondary building units) with organic linkers [8]. The rational choice of SBUs and linkers leads to a high degree of control over the pore dimensions of the crystalline framework, making possible the construction of microporous, as well as mesoporous, materials. From 2006 to 2010, Serre and co-workers reported high loadings of several APIs (ibuprofen, busulphan, doxorubicin, and others) inside highly-porous MOFs, such as MIL-100(Cr) [9] and MIL-100(Fe) [10]. In 2012, Serre [11] and Coronas [12] reported on the caffeine confinement inside Fe-MIL and zeolitic imidazolate (ZIF-8) frameworks, respectively. The examples dealing with the inclusion of nicotine in crystalline matrices are, instead, very limited [13,14,15], and this aspect adds value to the present study. 

**Figure 1:**
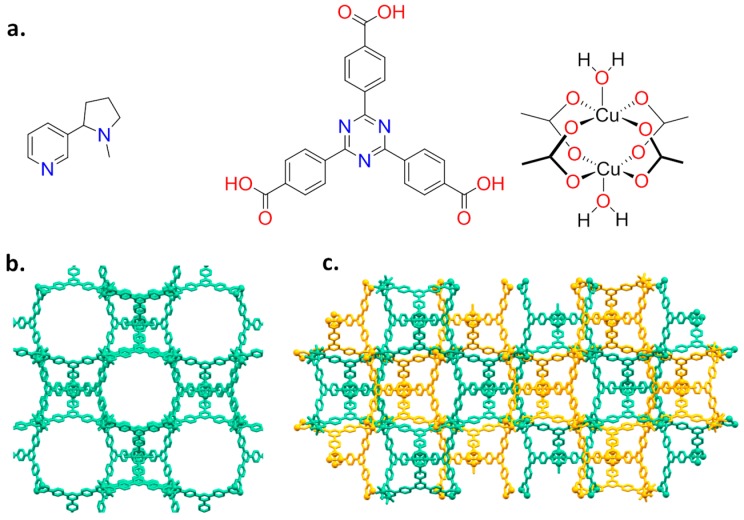
(**a**) Molecular structures of nicotine (left), TATB ligand (middle) and Cu paddle-wheel (right); and (**b**,**c**) the two isoreticular isomeric networks PCN-6′ and PCN-6. In PCN-6 the two interpenetrated frameworks are in different colours.

Nicotine has a bicyclic structure composed by a pyridine and a pyrrolidine ring, as depicted in Figure 1a.

The presence of a pyridine makes feasible the coordination of nicotine to a metal centre, as reported for Cu(II) [16,17]. As a host system, we then chose the mesoporous Cu(II)-based MOF PCN-6′ [18] (PCN stands for porous coordination networks), reported by Zhou in 2007. Here, each copper(II) ion is part of a paddle-wheel unit of the type Cu_2_(COO)_4_, where each carboxylate bridges the two metal ions. The coordination sphere of each metal is completed by a water molecule (Figure 1a). Replacement of the coordinated water molecule by the pyridine nitrogen of nicotine should lead to a stable guest upload. This, together with the high loading capacity deriving from the very large dimensions of the cavities featuring the framework of the chosen MOF, were considered good prerequisites for the construction of a material with high loading capacity. In particular, we were interested in obtaining the structural details on how nicotine interacts with the MOF walls. The anchoring of nicotine to copper atoms is expected to make the structural characterization of the nano-confined host-guest interaction easier, a particularly innovative aspect in the context of guest confinement in porous materials [19]. Here, we then report on the inclusion of nicotine into a mesoporous coordination network and the characterization of the derived material based on nuclear magnetic resonance (NMR), thermogravimetric (TGA), X-ray powder diffraction (XRPD), and single-crystal X-ray diffraction analysis (SC-XRD). 

## 2. Results and Discussion

### 2.1. Synthesis and Loading

**Figure 2:**
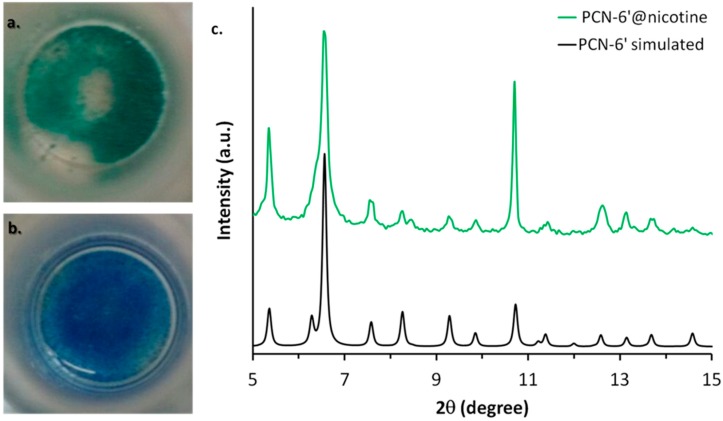
Colour change associated with the transformation of PCN-6′@dichloromethane (**b**) into PCN-6′@nicotine (**a**). (**c**) The comparison between the calculated XRPD trace of PCN-6′ (black) and the experimental one collected with PCN-6′@nicotine (green).

PCN-6′ can be considered the isostructural expanded analogue of classical microporous HKUST [20]. The structural motif of PCN-6′ comes from the connection of Cu(II) paddle-wheel nodes of the type [Cu_2_(COO)_4_∙2H_2_O] with the tritopic ligand 4,4′,4″-(1,3,5-triazine-2,4,6-triyl)tribenzoic acid (TATB), as reported in Figure 1a. This corresponds to the overall formula Cu_6_(H_2_O)_6_(TATB)_4_∙DMF∙12H_2_O (DMF stands for N,N′-dimethylformamide). As reported in Figure 1b, the resulting framework displays open square channels of 21.44 × 21.44 Å (Cu∙∙∙Cu distance) running along the three crystallographic axes *a*, *b*, and *c* (cuboctahedron symmetry). The channels give rise to internal mesoporous pores of 30.32 Å in diameter (Cu∙∙∙Cu distance). The turquoise octahedral crystals of PCN-6′ are formed under solvothermal conditions, reacting Cu(NO_3_)_2_∙3H_2_O with TATB in DMF at 80 °C. The synthesis was carried out in the presence of oxalic acid as templating agent [19] to avoid the formation of the corresponding interpenetrated phase known as PCN-6 [21]. The loading experiments were conducted by dipping the clean crystals of PCN-6′ into neat nicotine. The liquid nature of the guest avoided the use of a solvent and, hence, the resulting competition between the solvent and guest molecules in filling the MOF cavities. This, in turn, led to the isolation of a material composed only by the two components of interest, the host PCN-6′ and the guest nicotine. However, to reach this goal, an activation procedure prior to expose the MOF crystals to nicotine was necessary. This step was required to remove the DMF molecules filling the pores of the pristine MOF and the molecules of water coordinated to copper. The activation was performed following the solvent exchange protocol described by Zhou [18], which considers repeated soakings of the crystals in methanol (PCN-6@methanol) and then in dichloromethane (PCN-6@dichloromethane) prior to vacuum drying. The resulting purple phase was extremely hygroscopic and was handled under an inert atmosphere. Once put in contact with nicotine it turned light green in a few seconds. The chromatic change can be reasonably attributed to the entering of nicotine into the coordination sphere of Cu. The crystals were kept in contact with nicotine at room temperature for one week. After one week, the crystals were carefully removed from the vial and washed with acetone to remove the guest that was physically sorbed onto the crystals surface. Optical microscopy inspection evidenced that the crystals had maintained their habit during the uptake, without undergoing damage. Figure 2 shows evidence that the colour change provoked by nicotine uptake, from blue, corresponds to PCN-6′@dichloromethane, and to light green, corresponds to PCN-6′@nicotine.

The preservation of the crystalline framework of PCN-6′ was further confirmed by XRPD analysis, as shown by Figure 2c. The diffractogram calculated from the single crystal structure of PCN-6′ matches the diffractogram collected for PCN-6′@nicotine quite well. The crystals of PCN-6′@nicotine turned out to be significantly more stable towards moisture than those of pristine PCN-6′. These, in fact, once left in contact with air, quickly degraded with extensive cracking and fragmentation, a phenomenon which was not observed with the nicotine-containing material. MOF degradation is usually imputed to two main factors: hydrolytic processes at the expense of the carboxylate linkers, or fast desolvation of the cavities with a consequent collapse of the structure [22,23]. Hence, the higher stability found for PCN-6′@nicotine could be explained considering that filling the MOF cavities with a bulky thermally stable guest disfavours the adsorption of moisture and, at the same time, disfavours the loss of the guest. To obtain direct evidence of the nicotine uptake, the crystals of PCN-6′@nicotine were heated at 200 °C and the extruded vapours were analysed by mass spectrometry, by means of a direct-insertion-probe mass analyser. The resultant spectrum perfectly matched with the one expected for the guest (Figure S1, Supporting Information). The infrared spectroscopy confirmed the entering of the guest, as well. Aliphatic signals in the region between 2970 to 2870 cm^−1^ arising from the stretching of the pyrrolidine ring were quite visible only in the spectrum of PCN-6′@nicotine (Figure S2, Supporting Information). 

Several crystals of PCN-6′ loaded with nicotine were then analysed by single-crystal X-ray diffraction analysis, by means of synchrotron light radiation at 100 K. Unexpectedly, the structural resolution revealed the formation of PCN-6@nicotine as a concomitant phase, whose structural characterization is reported in the next paragraph. The structural characterization of PCN-6′@nictoine was not feasible due to severe twinning of the crystals. A detailed crystallographic characterization is reported in the next paragraph.

### 2.2. Structural Characterization of PCN-6@nicotine

The analysis has revealed the formation of PCN-6 as a concomitant phase of PCN-6′. Despite interpenetration, PCN-6 maintains a considerable pore dimension suitable for accommodating several guest molecules, which were characterized by structural analysis. 

SC-XRD measurements were performed with synchrotron light radiation in order to obtain high-resolution data and to understand whether the guest is ordered or partially organized inside the cavities. Diffraction experiments were conducted both by flash freezing of crystals at 100 K and by slow cooling (2 °C/min) from room temperature to 100 K, in an attempt to equilibrate the guest molecules towards an ordered disposition inside the cavities. No significant differences were obtained in the final electron density maps by the two cooling protocols.

The analysis conducted on several crystals revealed the presence of two different crystalline phases: one cubic and one trigonal (the unit cell data of the two phases are reported in Table S2, Supporting Information), corresponding to the inclusion products of PCN-6′ and PCN-6, respectively. The latter is the interpenetrated counterpart of PCN-6′, and the two are catenation isomers. The structure of pristine PCN-6 (space group R-3m) is described by two identical interpenetrated nets of PCN-6′, translationally displaced by one fifth of the c axis along the [001] direction [21]. The translation is due to π-π interactions between two face-to-face stacked TATB ligands. It was noted that soaking with nicotine partially damaged the crystals of PCN-6′, therefore, it was not possible to collect data allowing the identification of nicotine molecules in the structure. Conversely, despite interpenetration, PCN-6 maintains a considerable pore dimension suitable for inclusion of molecular guests and crystals remained good enough to ensure a satisfactory and complete data collection. This is probably due to better mechanical properties of interpenetrated MOF crystals. The presence of chiral nicotine inside the structure lowers the symmetry of PCN-6@nicotine to R32.

**Figure 3:**
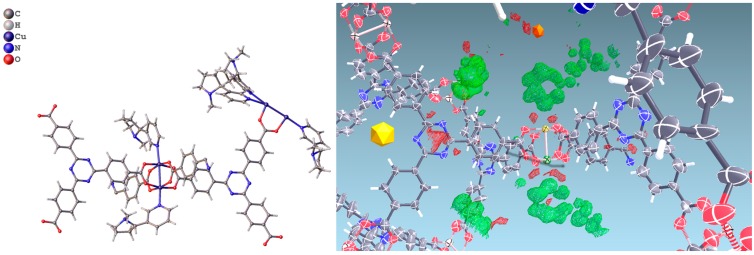
(**Left**) Asymmetric unit of the structure of PCN-6@nicotine, showing four guests coordinated to the copper atoms of the paddlewheel SBU; one guest is disordered on two positions; (**Right**) Difference Fourier map (drawn at the 2.5 sigma level) showing the electron density of two ordered coordinated guests.

In PCN-6@nicotine each Cu atom coordinates one nicotine molecule, evident in the difference Fourier map (Figure 3), for a total of four independent nicotine molecules in the final map. The final electron density map of PCN-6@nicotine was modelled by fitting a rigid molecular model of nicotine into the residuals of the difference Fourier map, adapting the reciprocal orientation of the two rings to the observed shape of the electron density cloud. In one site two disordered orientations are present, respectively occupied by 60% and 40%. The thermal parameters of the pyrrolidinic rings are high to testify the mobility or displacive disorder of the guests inside the pores, even at 100 K (Figure S5, Supporting Information).

**Figure 4:**
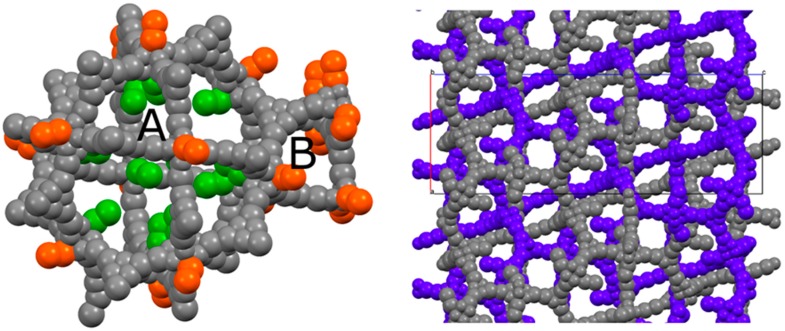
(**Left**) Schematic view of the pores of the simple PCN-6@nicotine net: two cavities, A (large, cubo-octahedral) and B (small, tetrahedral), are evident; the MOF skeleton is schematized in grey with pseudo-atoms, nicotine molecules are represented by the centre of mass of the rings, in green the 12 pointing inside the A cavity, and in orange those belonging to the neighbouring pores. (**Right**) The two interpenetrated nets of PCN-6@nicotine are schematized with the same representation, showing that pores are partitioned, but not completely filled.

This particular type of coordination has also been observed in the structure of a cluster which is extremely similar to the one featuring PCN-6 and PCN-6′, being formed by tetramethacrylate ligands in place of carboxylate ligands [17]. In addition to these molecules of the guest stabilized by coordinative bonds, the structure potentially contains many other nicotine molecules free to arrange in the MOF pores. Although it was not possible to model them (Figure S6, Supporting Information), from the refinement it was possible to derive a structural hypothesis for the loading of nicotine in PCN-6@nicotine. Looking at the coordination sites present in the frameworks, the parent framework of PCN-6′ exhibits two cavities, one larger cubo-octahedral, and one smaller tetrahedral; twelve coordination sites point inside the former, none inside the latter. The same primary skeleton is found in PCN-6@nicotine, Figure 4, but interpenetration reduces the inner available space of the pores and splits the larger cavity. 

**Figure 5:**
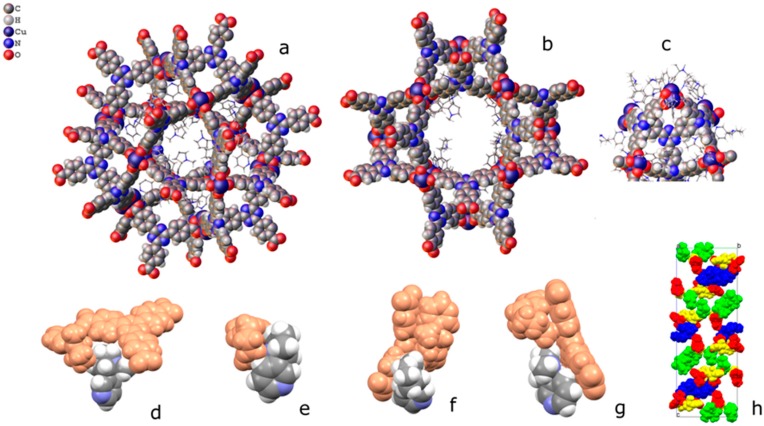
Packing of coordinated nicotine molecules in PCN-6@nicotine. (**a**,**b**) two orientations of pore A, with coordinated nicotine protruding in the cavity; (**c**) pore B; (**d**–**g**) contacts of the four coordinated nicotine molecules with other guests and with the MOF skeleton; and (**h**) packing of the four nicotine molecules (evidenced in four colours) inside the structure, with the MOF skeleton removed.

Coordinated nicotine molecules protrude into the cavities from the inner surface of the A pores (Figure 5) and are partially in contact with each other, or with the MOF skeleton.

**Figure 6:**
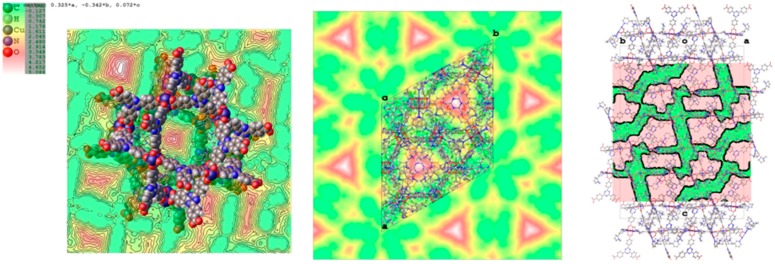
Maps of the voids in PCN-6@nicotine, where the unstructured residual electron density has been detected. (**Left**) Void present in pore A; (**centre**) map of the voids in the unit cell projected down the *c* axis; (**right**) mask of the accessible voids in the unit cell mapped down [110].

The coordination of nicotine molecules to the copper atoms does not completely fill the volume of the cavities (Figure 6), resulting in a total empty volume per unit cell of 39,000 Å^3^, accommodating 11,000 electrons, as estimated by the Fourier map.

By considering a loose packing efficiency of 50% of nicotine inside the cavities, it can be roughly estimated that the unit cell contains about 100 nicotine molecules (molecular volume = 180 Å^3^, 88 electrons) not directly coordinated to the copper atoms, resulting in approximately 9000 electrons, which is in reasonable agreement with the residual 11,000 electrons estimated with the squeeze procedure in Olex2 (v1.2.8) [24]. It must be noted that this refers to the total empty volume available, which consists of symmetry-related copies of three connected pores of different shape and volume, centred respectively, at (0, 0, 0), (0, 0, 0.15), and (0, 0, 0.38), with volumes of approximately 2000 Å^3^ for the first two, and 1000 Å^3^ for the third one. The largest sphere that can be accommodated has a radius of 5.90 Å, and the cavities communicate by channels with a window of 2.7 Å along *a* and *b*, and of 1.50 Å along *c*.

### 2.3. Characterization of Nicotine-Containing Material

On one hand, the proposed templating strategy expected to result exclusively in PCN-6′ partly failed, with the formation of a parasite fraction of the interpenetrated phase PCN-6. A mechanical separation of the two phases was barely impossible, as the crystals have the same size, colour, and morphology. Furthermore, the discrimination of the two phases by XRPD analysis was unfeasible since peak positions are coincident in the two diffractograms calculated from the respective single-crystal structures (Figure S4, Supporting Information). However, the structural analysis performed on the parasite interpenetrated phase shows, very well, that nicotine is captured efficiently by this material. 

**Figure 7:**
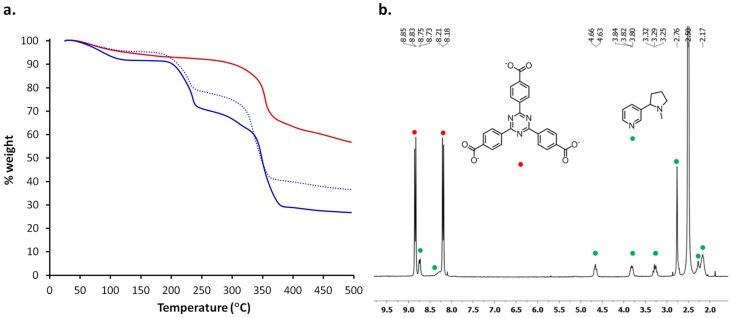
(**a**) TGA traces of PCN-6′ after activation (red), PCN-6′@nicotine (blue) and PCN-6′@nicotine after six months (dotted blue); (**b**) ^1^H NMR spectrum of PCN-6′@nicotine recorded after dissolution of the crystal in a TFA-d/DMSO-d_6_ mixture. The signals of the ligand TATB are indicated with red bullets, while the signals belonging to nicotine are indicated with green bullets.

The quantification of the amount of trapped nicotine was based on TGA and NMR analysis, considering PCN-6′ as the predominant crystalline phase. Then, hereinafter, the acronym PCN-6′ will refer to the mixture of PCN-6′ contaminated by traces of PCN-6. The thermograms are collected in Figure 7.

The TGA trace of activated PCN-6′ does not display any weight loss until MOF decarboxylation, which occurs at 320 °C (the red trace in Figure 4a). On the contrary, the TGA trace of PCN-6′@nicotine (the blue trace in Figure 7a) shows a 21% weight loss in the range 180–240 °C, a second weight loss between 250 and 300 °C and, finally, decarboxylation occurring at 320 °C. The second guest extrusion is partly concomitant with the decarboxylation of the MOF, and then difficult to quantify. We assume that the first loss is attributable to the departure of the molecules of nicotine filling the cavities of the host framework and not interacting significantly with the walls of the MOF. The second inflection is, instead, mainly ascribable to the detachment of the coordinated nicotine or nicotine molecules interacting with the MOF walls through dispersive forces. This is consistent with the reported thermal behaviour of nicotine containing metal complexes, which shows the nicotine release between 280 and 400 °C [25].

The high stability of the host/guest material was highlighted by a TGA analysis repeated after having stored crystals of PCN-6′@nicotine for six months in air. A guest release corresponding to 17% weight loss in the temperature range 180–240 °C was observed (dotted curve in Figure 7a), meaning that only a minimal fraction of the guest was lost during storage.

In order to better quantify the amount of trapped nicotine, we directed our attention to NMR spectroscopy. Despite the paramagnetic character of Cu(II), NMR spectroscopy was believed to be a useful technique for a reliable guest quantification [26]. 

Crystals of PCN-6′@nicotine were digested in a mixture of TFA-d/DMSO-d_6_. The resulting clear solution was first analysed by ^13^C{^1^H}-NMR spectroscopy, to check the feasibility of the technique. In the interval 180–120 ppm the peaks belonging to TATB were visible (Figure S7, Supporting Information). The pyridine signals were not well resolved, being fused in a broad signal. The line broadening is likely due to the presence of the paramagnetic metal ion. Moving toward the aliphatic carbons, we can recognize three signals at 56.2, 38.6, and 31.2 ppm, respectively, which can be assigned to CH_2_ carbons of pyrrolidine. Finally, at 21.9 ppm the CH_3_ peak is found [27]. The ^1^H-NMR spectrum of the solution was subsequently collected (Figure 7b). The spectrum showed two sharp doublets corresponding to the 12 aromatic protons of TATB (8.84 and 8.20 ppm). Pyridine gave rise to a broad doublet centred at 8.74 ppm belonging to the proton in para form with respect to nitrogen. The signal arising from the two protons ortho form to nitrogen were not visible, pointing out that in the deuterated solution pyridine is still engaged in coordination with the metal. The broad signal centred at about 8.28 ppm and partly fused with the doublet of TATB can be assigned to the proton in meta form to nitrogen. In the aliphatic region, in the interval of 2.1–4.7 ppm, the three broad multiplets and the two broad signals can be assigned to CH and CH_2_ moieties of pyrrolidine ring, respectively, whereas the broad singlet at 2.76 ppm derives from the methyl protons (NCH_3_).

For a reliable quantification of nicotine, we chose the methyl group of the pyrrolidine ring as a probe. Its peak multiplicity corresponds to a higher signal-to-noise ratio, thus making the quantification analysis more consistent. The unambiguous assignment of the methyl signal was further confirmed by a COSY experiment: no cross peaks were observed for the NCH_3_ signal (Figure S8, Supporting Information).

From the crystalline structure of PCN-6′ one can see that each pore is delimited by eight molecules of TATB, corresponding to 96 aromatic protons and twelve Cu paddle-wheel units. For this reason, a normalized value of 48 protons was attributed to each doublet centred at 8.84 and 8.20 ppm of Figure 7b. Hence, the integration area corresponding to the singlet belonging to the methyl group of the pyrrolidine ring corresponds to 20 molecules of nicotine included in each pore of the MOF. This leads to the unit formula Cu_6_(TATB)_4_∙10nicotine for PCN-6′@nicotine. This would correspond to a 42% weight of nicotine included in PCN-6′@nicotine. This is a value which overestimates the amount of trapped guest with respect to the one defined from TGA analysis up to 240 °C, corresponding to 21%. However, it must be recalled that a fraction of nicotine is thermally extruded just before, and during, the collapse of the framework occurring during the decarboxylation process. This hypothesis was confirmed subjecting a sample of PCN-6′@nicotine to TGA up to a temperature of 240 °C, and subsequently recording the ^1^H-NMR spectrum of the residue. The resulting spectrum revealed the presence of a residual 17% weight of nicotine (Supporting Information, Figure S9), leading to a total amount of nicotine determined by a TGA analysis of 38%. This value is in good agreement with the one determined by NMR analysis.

Later on, we became interested in studying the loading capacity of PCN-6′ from a nicotine-containing solution. The kinetic uptake profile was determined by monitoring the concentration decrease of a dichloromethane solution of nicotine with time. The experiment was conducted at room temperature. The starting concentration of the solution was 3.68 × 10^−3^ M and dropped to 1.74 × 10^−5^ M in 11 days through a first-order kinetic profile (Figure S10, Supporting Information). After 11 days, the concentration did not vary significantly, indicating equilibration of the solution. The nicotine concentration drop was around 240 times the starting value, corresponding to the almost complete removal of nicotine from the dichloromethane solution. Most of the loaded nicotine (77%) was included within the first three days, while in the remaining period the uptake was much slower. This behaviour highlights the high affinity of the host framework towards nicotine.

## 3. Materials and Methods 

TATB was synthesized with a slightly modified procedure with respect to the one reported in the literature [18]. (−)-Nicotine (99%) and all the other reagents and solvents were used as received. 

^1^H-NMR and ^13^C{^1^H}-NMR spectra were recorded on 400 MHz and 300 MHz Bruker instruments (Billerica, MA, USA). Chemical shifts are reported in ppm relative to the solvent residual peak of (CD_3_)_2_SO (δH 2.50, δC 39.5). TFA-*d* is used for the digestion experiments. 

EI(+)-MS spectra were collected with an ionising voltage of 70 eV by means of a Thermo DSQ II spectrometer equipped with a single-quadrupole analyser (Waltham, MA, USA). The analyses were conducted on solid samples by means of a DIP probe (direct insertion probe, flash thermolysis at 350 °C). 

IR spectra were obtained with a Thermo Scientific Nicolet 5PCFT-IR-ATR spectrometer (diamond crystal) in the 4000–400 cm^−1^ interval. 

TGA analyses were performed on a Mettler Toledo TGA/DSC1 instrument (sample mass approx. 5–10 mg) at a heating rate of 10 °C∙min^−1^ in a temperature range from 25–500 °C. The measurement was performed at atmospheric pressure under nitrogen, 80 mL∙min^−1^ (Columbus, OH, USA). 

UV-VIS spectra were collected on a UV-VIS Bio Evolution Thermo scientific 260 spectrophotometer and all the concentrations were calculated using the maximum value of absorbance (nicotine λmax = 263 nm). An initial solution (3.68 × 10^−3^ M) of nicotine in anhydrous CH_2_Cl_2_ (5 mL) was inserted in a flask along with activated PCN-6′ (6.7 mg). Aliquots of 50 μL of the supernatant solution have been taken and properly diluted to 1/100 in order to be in the range of the calibration curve (Waltham, MA, USA). 

X-ray single-crystal measurements were performed at *Elettra Sincrotrone* (Trieste, Italy) on beamline XRD1 [28] at 100 K under cold nitrogen flux. The beamline spectra (produced by a NdBFe multipole wiggler) has been monochromatized to 17.71 KeV (0.700 Å) through a Si (111) double crystal monochromator and focused to obtain a beam size of 0.2 × 0.2 mm FWHM at the sample (photon flux 10^12^–10^13^ ph/sec). 

Soaked PCN-6′/PCN-6 crystals were taken directly from nicotine and mounted with cryoloops (0.05–0.3 mm). An annealing trial was performed on mounted crystal, dropping the temperature from r.t. to 100 K, with a cooling rate of 2 °C min^−1^. Diffraction data were indexed, integrated, and scaled using CrysAlis software (v38.43) [29]. The PCN6@nicotine structure was solved by direct methods using SHELXS97 [30] and refined by full-matrix least-squares on all F^2^ using SHELXL implemented in Olex2 (v. 1.2.8.) [24]. Anisotropic displacement parameters were refined except for hydrogen atoms. The final Flack parameter, unsatisfactory to assess the absolute configuration, reflects the poor diffracting power of the chiral guest compared to the centric MOF framework. The absolute configuration of nicotine was known and used in modelling. Nicotine molecules were constrained to their ideal geometry and refined as rigid bodies. SHELXE [31] was used to inspect the electron density maps. Residual electron density in the pores was estimated by the mask procedure in Olex2 [32], and the final refinements performed by taking into account the unstructured density (final R = 5.18) and without any solvent mask (R = 11.23) were compared. Geometric characterization of the voids has been performed with Olex2 [24] and MERCURY (v. 3.9) [33]. The software of Cambridge Crystallographic Data Centre CCDC [34] has been extensively used for the comparison with known structures. Table S1 (Supporting Information) reports crystal data and refinement results for PCN-6@nicotine. Final masked data have been deposited and CCDC 1555580 contains the supplementary crystallographic data for this paper.

### 3.1. ATB-4,4′,4″-(1,3,5-Triazine-2,4,6-Triyl)Tribenzoic Acid

To a 250 mL round bottomed flask acetic acid (40 mL), 2,4,6-tri-p-tolyl-1,3,5-triazine (1.5 g, 4.3 mmol) and H_2_SO_4_ (2.5 mL) were added, obtaining a pale yellow mixture. After cooling to 0 °C with an ice bath, CrO_3_ (3.9 g, 39 mmol, 9 equivalents) and acetic anhydride (2.4 mL) were added to provide a dark brown solution. Further 20 mL of acetic acid was added and the reaction was stirred at room temperature overnight. Then, the reaction was quenched with distilled water (200 mL) obtaining the formation of a very fine product suspension. The resulting mixture was separated by centrifugation and carefully washed by adding water until no further precipitate formation was detected (deep green supernatant), observed as a pale greenish solid. The isolated solid was carefully washed with water, followed by acetone, and finally dried overnight under vacuum. TATB was obtained as an off-white solid (1.31 g, 2.96 mmol, y = 86%).

### 3.2. Recrystallization Procedure

In a 100 mL round-bottomed flask TATB (1.31 g, 2.96 mmol) was dissolved under magnetic stirring in DMF (65 mL) at 80 °C. The limpid pale green solution was cooled to r.t. and stored at −20 °C overnight. Crystallized TATB was isolated by vacuum filtration with a glass septum (1.07 g, 2.42 mmol, yield = 82%). 

^1^H-NMR (300 MHz, DMSO-d_6_, 25 °C): *δ* 13.30 (br, COO*H*, 3H), 8.65 (d, C*H*Ar, *J*= 8.2 Hz, 6H), 8.08 (d, C*H*Ar, *J* = 8.2 Hz, 6H) ppm; ^13^C-NMR (75 MHz, DMSO-d_6_, 25 °C): *δ* 170.4, 166.8, 138.6, 134.6, 129.7, 128.7 ppm.

### 3.3. Synthesis of PCN-6′

In a 16 mL Pyrex tube TATB (20 mg, 0.044 mmol), Cu(NO_3_)_2_∙3H_2_O (52 mg, 0.214 mmol), and oxalic acid dihydrate (4 mg, 0.030 mmol) were added to DMF (3 mL). Then, the tube was tightly capped and heated at 80 °C in an oil bath for 48 h. Note that after a few hours the formation of copper oxalate as a very fine pale blue powder was observed. Its nature was confirmed by FT-IR spectroscopy (see Supporting Information, Figure S3) [35]. The crystals of PCN-6′ could be conveniently separated from copper oxalate by repeated washings with DMF. As reported in the main text, single-crystal X-ray analysis conducted on several crystals containing nicotine revealed the presence of the concomitant non-interpenetrated phase PCN-6. The separation of the two phases and the quantification of PCN-6 were not feasible.

### 3.4. Activation and Soaking with Neat Nicotine

PCN-6′ samples were activated following a solvent exchange protocol: as-synthesized crystals were soaked in methanol for 24 h, then the solvent was discarded. Fresh methanol was subsequently added, and the crystals were allowed to soak for an additional 24 h. The same procedure was repeated with dichloromethane. After removal of dichloromethane the sample was transferred in a Schlenk tube and dried overnight under vacuum (<10^−3^ Torr) at room temperature. All these steps were accompanied by drastic colour changes of the crystals, from turquoise (native crystals containing DMF), to light-blue, to blue, and finally to purple. This phase must be stored under nitrogen to avoid immediate rehydration.

After the evacuation, 10 mg of activated PCN-6′ crystals were dipped in neat nicotine (1 mL) under nitrogen flux. The vial was then tightly capped and the soaking was performed under static conditions. After one week, nicotine was removed and crystals were carefully washed with acetone (1 mL) two times, then dried under vacuum to provide PCN-6′@nicotine as green crystals.

## 4. Conclusions

In this work, we describe the entrapment of the very popular liquid compound, nicotine, into a mesoporous metal organic framework. Trapping was performed by direct immersion of activated crystals of the MOF into neat nicotine. The uptake process occurred with complete retention of crystallinity, as evidenced by XRPD analysis. Importantly, in the case of PCN-6@nicotine, a single-crystal to single-crystal transformation occurred, as evidenced by single-crystal X-ray analysis. This technique showed the coordination of nicotine to the paddle-wheel units composing the MOF framework. A careful modelling of the electron density map contained in the MOF cavities led to the definition of the Cu-pyridine interaction, which can then be considered the process which triggers the entire uptake process. The joint application of NMR and TGA analyses outlined a very efficient guest uptake. From the combination of these two techniques, a loading higher than 40 wt % was established. This result is in line with other massive guest trappings reported in the literature for other porous MOFs, such as (Fe)MIL-100 (e.g., 50 wt % caffeine loading) [11]. 

## Supplementary Materials

The following are available online at www.mdpi.com/1996-1944/10/7/727/s1, MS, IR, NMR, UV-VIS spectra; XRPD traces, and structural images.
