# Supplementary material for: Coordination Driven Capture of Nicotine Inside a Mesoporous MOF
Source: Materials (Basel). 2017 Jun 30;10(7):727. doi: 10.3390/ma10070727 (PMC5551770; doi:10.3390/ma10070727)
Supplement: Supplementary file 1 [file materials-10-00727-s001.pdf]

Supplementary Materials: Coordination driven capture of nicotine inside a mesoporous MOF

Davide Balestri [1], Davide Capucci [1], Nicola Demitri [2], Alessia Bacchi [1] and Paolo Pelagatti [1,*]

Table of contents	Page
DIP-EI analysis of MOF@guest	S2
FT-IR spectra	S2-S3
X-ray diffraction	S4-S6
^{1}H-NMR digestion for MOF@guest	S6-S7
UV-VIS analysis	S8

Mass spectrometry

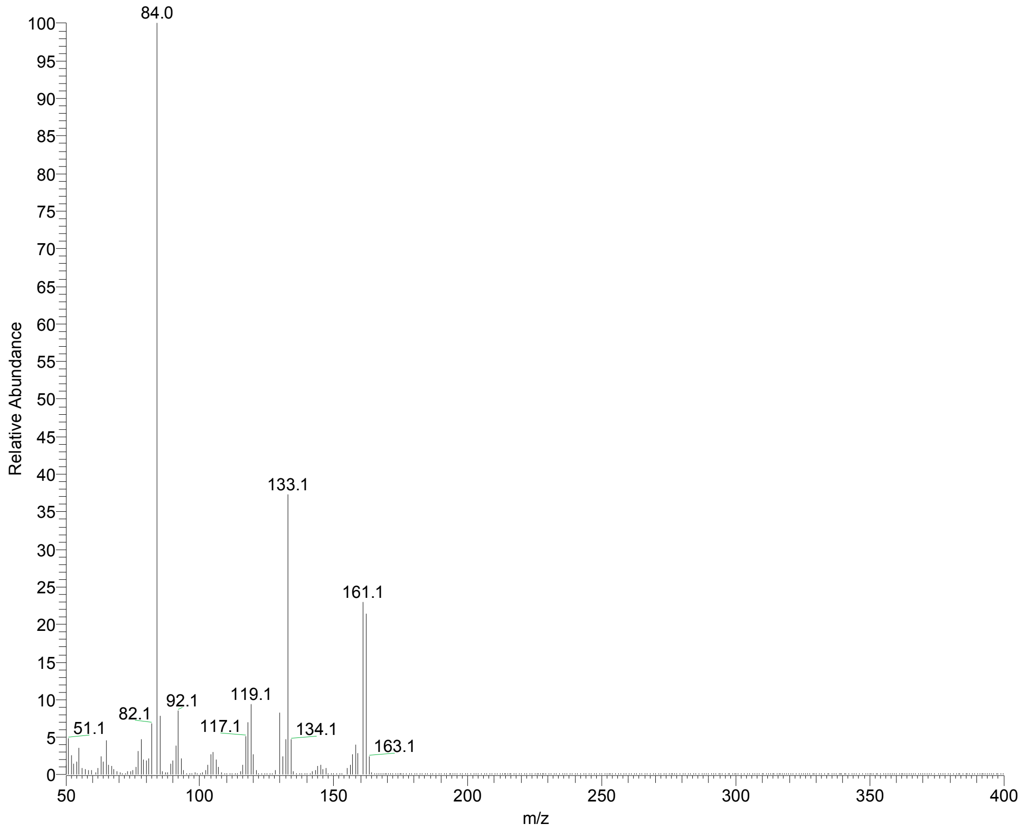

Figure S1. **(**DIP)MS-EI(+) spectrum of nicotine thermally extruded from crystals of PCN-6′@nicotine at 200 °C. Diagnostic signals: m/z = 161.1, 133.1, 119.1, 92.1, 84.0.

FT-IR spectroscopy

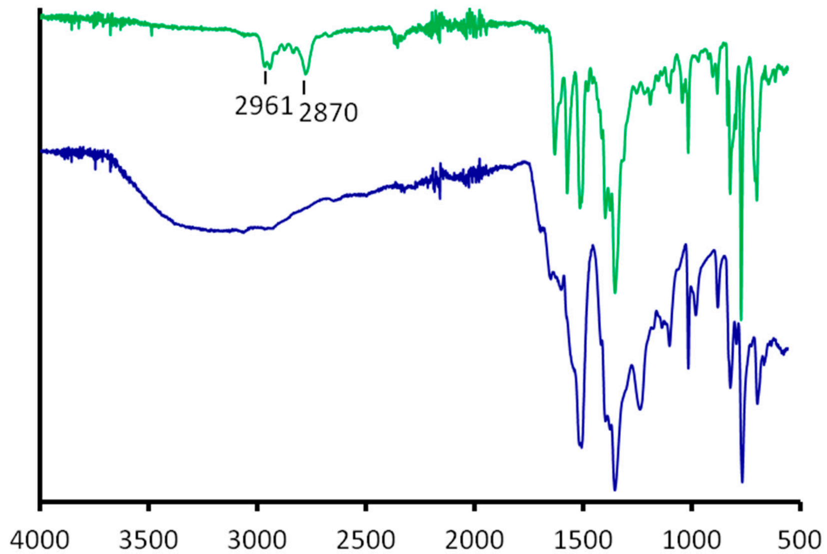

Figure S2. Comparison between FT-IR spectra of pristine PCN-6′ (**blue**) and PCN-6′@nicotine (**green**)

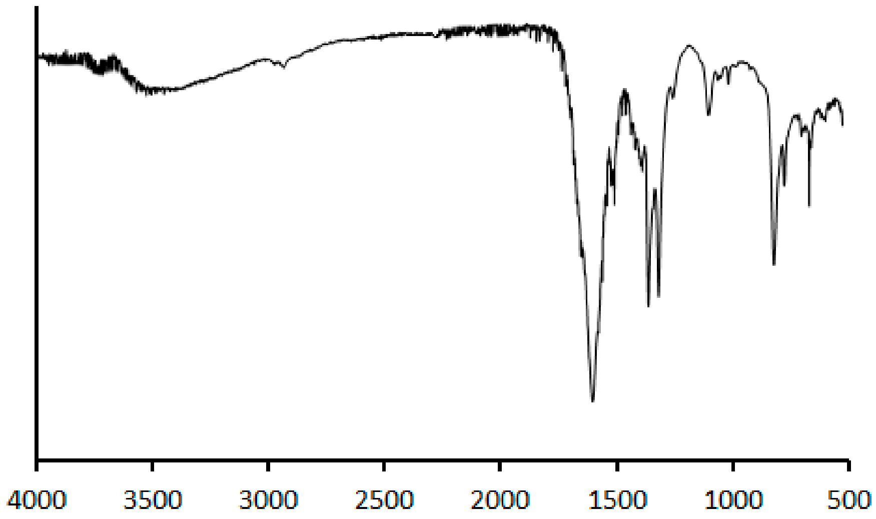

Figure S3. FT-IR spectrum of copper(II) oxalate formed during the synthesis of PCN-6′ [1].

X-ray diffraction

Table S1. Crystal data and structure refinement for PCN6@nicotine with the solvent mask procedure.

Empirical formula	$C_{208}H_{172}Cu_8N_{32}O_{32}$
Formula weight	4140.09
Temperature/K	100.15
Crystal system	trigonal
Space group	R32
a/Å	33.093(1)
b/Å	33.093(1)
c/Å	79.840(2)
α/°	90
β/°	90
γ/°	120
Volume/Å^3	75722(5)
Z	9
ρ_{calc} g/cm^3	0.817
μ/mm^{-1}	0.521
F(000)	19188.0
Radiation/ Å	synchrotron (λ = 0.700)
2Θ range for data collection/°	1.486 to 45.768
Index ranges	$-36 \le h \le 36$, $-36 \le k \le 32$, $-88 \le l \le 88$
Reflections collected	102014
Independent reflections	24158 [R_{int} = 0.0774, R_{sigma} = 0.0604]
Data/restraints/parameters	24158/1164/1130
Goodness-of-fit on F^2	1.018
Final R indexes [I>=2σ (I)]	R_1 = 0.0518, wR_2 = 0.1456
Final R indexes [all data]	R_1 = 0.0848, wR_2 = 0.1659
Final ΔF max/min/e Å^{-3}	0.33/−0.36
Flack parameter	0.375(13)

Table S2. Comparison between the unit cell parameters for pristine PCN-6′ and PCN-6.

**Parameters**	**PCN-6′**	**PCN-6**
Space group	*Fm-3m*	*R-3m*
a (Å)	46.636	32.968
b(Å)	46.636	32.968
c (Å)	46.636	80.783
α (°)	90	90
β(°)	90	90
γ(°)	90	120
Volume	101432	76039

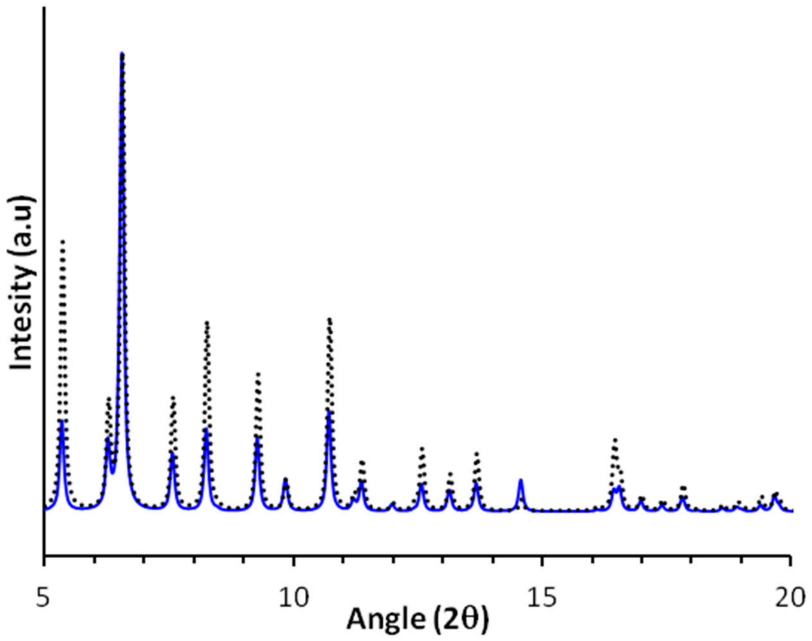

Figure S4. Comparison between the calculated XRPD traces of PCN-6′ (solid blue line) and PCN-6 (black dotted line).

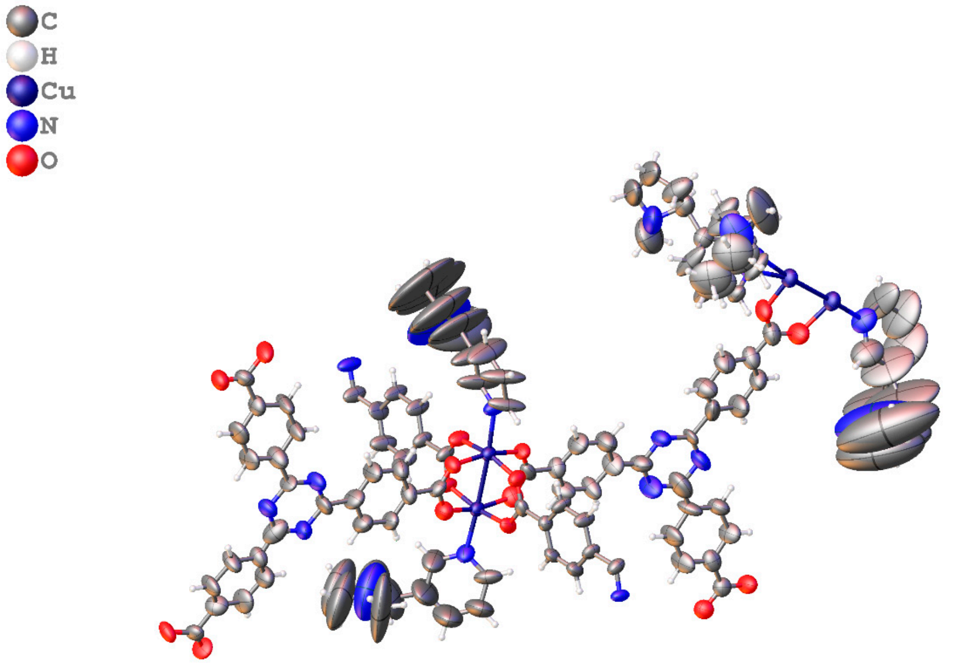

Figure S5. Anisotropic thermal displacement parameters of the coordinated nicotine molecules shows high mobility or displacive disorder in PCN-6@nicotine.

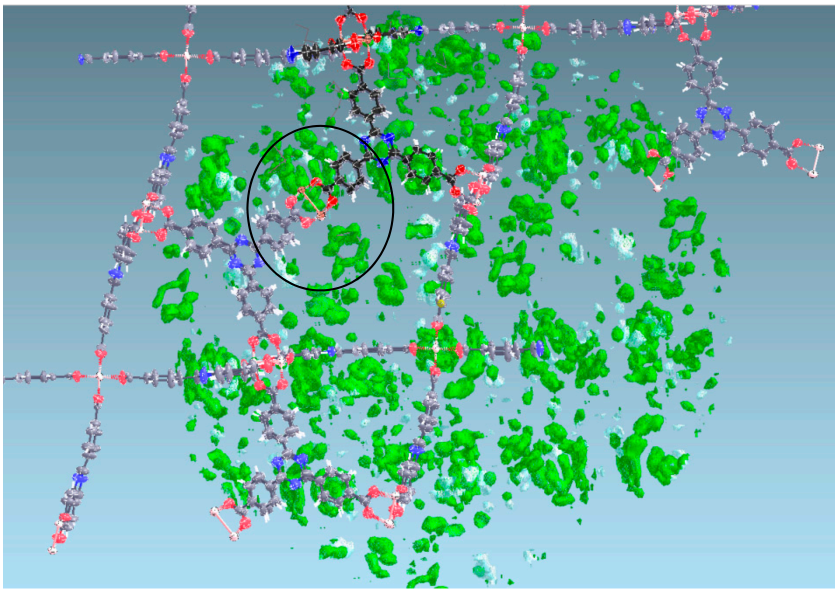

Figure S6. Final residual difference electron density map in the pores of PCN-6@nicotine, showing no structured electron density in the cavity besides the coordinated nicotine molecules (circled).

NMR spectroscopy

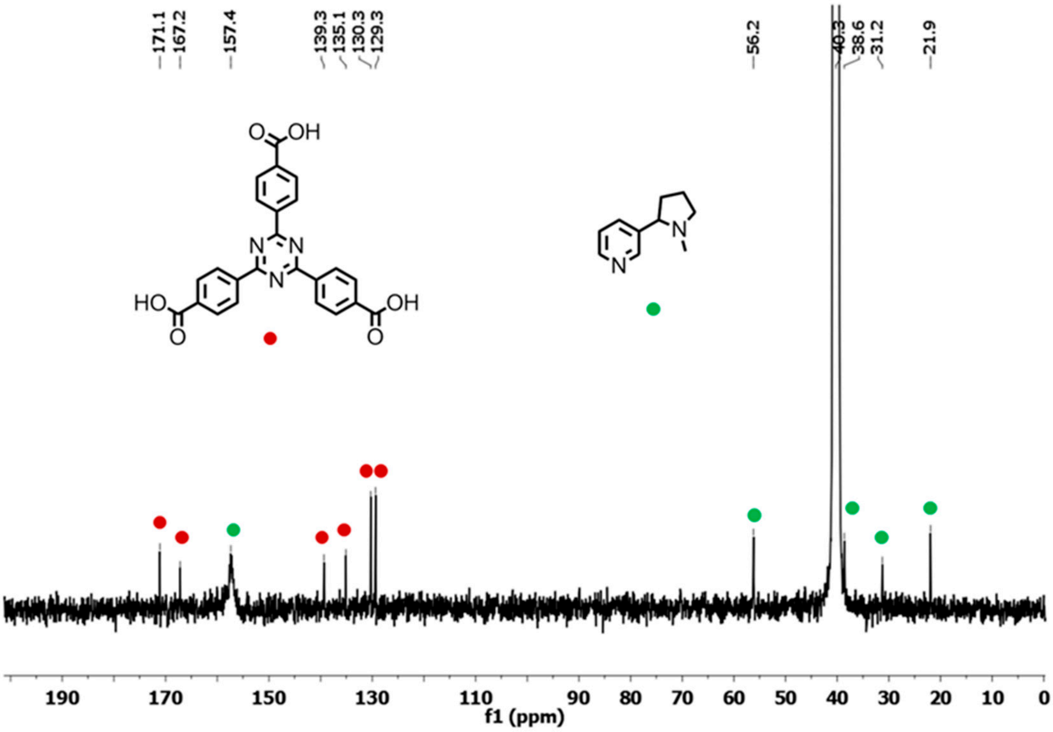

Figure S7. $^{13}C\{^{1}H\}$-NMR spectrum (100 MHz, DMSO-d⁶/TFA-d, 25°C) of digested PCN-6′@nicotine crystals

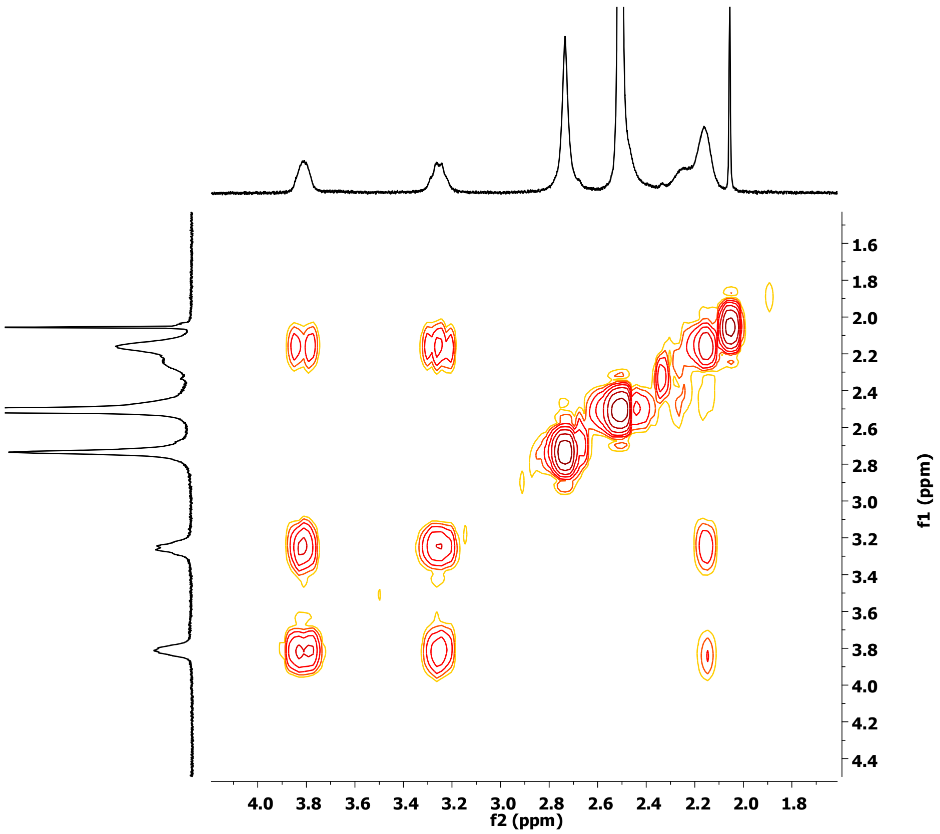

Figure S8 Expansion of the aliphatic region of the COSY spectrum of digested PCN-6´@nicotine crystals (TFA-d/DMSO-d⁶).

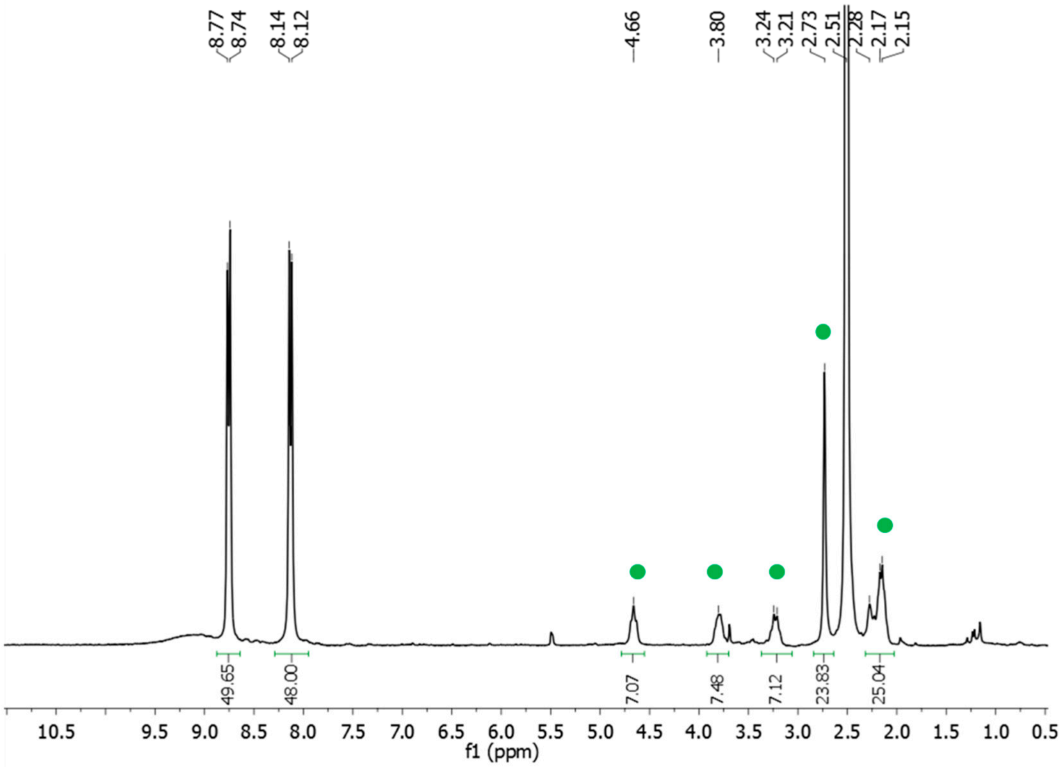

Figure S9: ^{1}H-NMR (300 MHz, DMSO-d⁶/TFA-d, 25°C) spectrum of PCN-6'@nicotine after heating up to 240°C: nicotine protons are still present (green ballets).

UV-Vis spectroscopy

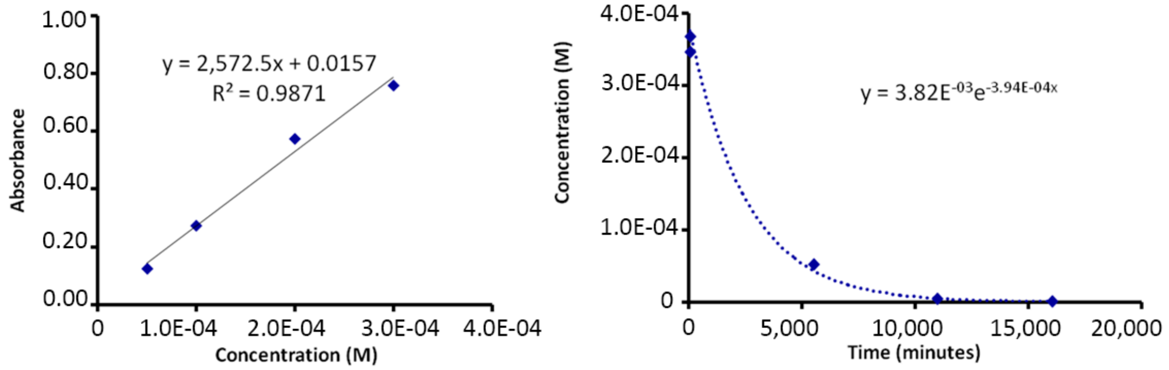

Figure S10: **Left**: calibration curve for UV-VIS analysis obtained using dichloromethane solutions of known concentrations of nicotine. **Right**: exponential plot showing the decrease of nicotine concentration over time for the uptake experiment conducted in a dichloromethane solution of nicotine

References

1. Edwards, H.G.M.; Farwell, D.W.; Rose, S.J.; Smith, D.N. Vibrational spectra of copper(II) oxalate dehydrate, $CuC_2O_4{\cdot}2H_2O$, and dipotassium bis-oxalato copper(II) tetrahydrate, $K_2Cu(C_2O_4)_2{\cdot}4H_2O$. *J. Mol. Struct.* **1991**, *249*, 233–243, doi:10.1016/0022-2860(91)85070-J.
